# Development of a Biosafety Level 1 Cellular Assay for Identifying Small-Molecule Antivirals Targeting the Main Protease of SARS-CoV-2: Evaluation of Cellular Activity of GC376, Boceprevir, Carmofur, Ebselen, and Selenoneine

**DOI:** 10.3390/ijms25115767

**Published:** 2024-05-25

**Authors:** Yasunori Fukumoto, Noriyuki Suzuki, Reina Hara, Yu-ki Tanaka, Yasumitsu Ogra

**Affiliations:** Graduate School of Pharmaceutical Sciences, Chiba University, Chiba 260-8675, Japan; n-suzuki@chiba-u.jp (N.S.); yu-ki.tanaka@chiba-u.jp (Y.-k.T.); ogra@chiba-u.jp (Y.O.)

**Keywords:** SARS-CoV-2, Mpro, GC376, boceprevir, carmofur, ebselen, selenoneine, binding energy calculation

## Abstract

While research has identified several inhibitors of the main protease (Mpro) of SARS-CoV-2, a significant portion of these compounds exhibit reduced activity in the presence of reducing agents, raising concerns about their effectiveness in vivo. Furthermore, the conventional biosafety level 3 (BSL-3) for cellular assays using viral particles poses a limitation for the widespread evaluation of Mpro inhibitor efficacy in a cell-based assay. Here, we established a BSL-1 compatible cellular assay to evaluate the in vivo potential of Mpro inhibitors. This assay utilizes mammalian cells expressing a tagged Mpro construct containing N-terminal glutathione *S*-transferase (GST) and C-terminal hemagglutinin (HA) tags and monitors Mpro autodigestion. Using this method, GC376 and boceprevir effectively inhibited Mpro autodigestion, suggesting their potential in vivo activity. Conversely, carmofur and ebselen did not exhibit significant inhibitory effects in this assay. We further investigated the inhibitory potential of selenoneine on Mpro using this approach. Computational analyses of binding energies suggest that noncovalent interactions play a critical role in facilitating the covalent modification of the C145 residue, leading to Mpro inhibition. Our method is straightforward, cost-effective, and readily applicable in standard laboratories, making it accessible to researchers with varying levels of expertise in infectious diseases.

## 1. Introduction

Since its emergence in 2019, severe acute respiratory syndrome coronavirus 2 (SARS-CoV-2), the virus responsible for the global COVID-19 pandemic, has necessitated the urgent development of therapeutic interventions. One promising strategy targets the main protease (Mpro), a key enzyme encoded by the viral genome. Mpro is a 3-chymotrypsin-like cysteine protease essential for viral replication, making it a focus for research on small-molecule antivirals. Nirmatrelvir, recently approved by the U.S. Food and Drug Administration [1], and ensitrelvir, approved in Japan [2], represent some of the first approved antiviral drugs targeting the Mpro of SARS-CoV-2. In addition to these clinically approved drugs, research efforts have identified several other potential therapeutic inhibitors of Mpro activity, including ebselen, disulfiram, carmofur, PX-12, tideglusib, and shikonin [3]. GC376, originally developed as a broad-spectrum antiviral [4], and boceprevir, a known inhibitor of the hepatitis C virus protease [5], have also been shown to inhibit SARS-CoV-2 by targeting Mpro [6].

One study has raised concerns about the effectiveness of some Mpro inhibitors in vivo, particularly in environments with reducing agents [7]. Ebselen, carmofur, and PX-12 achieve Mpro inhibition through covalent modification of the catalytic cysteine residue [3]. This covalent modification involves disulfide or selenium–sulfide bonds, raising concerns about potential cleavage by reducing agents present in vivo, potentially limiting their effectiveness within living systems. Indeed, several Mpro inhibitors, including ebselen, carmofur, disulfiram, PX-12, tideglusib, and shikonin, exhibit reduced activity in the presence of the reducing agent dithiothreitol (DTT) [7]. Nirmatrelvir also modifies the catalytic cysteine residue of Mpro [8]. This finding is concerning because cellular environments contain millimolar concentrations of glutathione, another reducing agent. Glutathione is present in all tissues and regulated by cellular redox systems to eliminate reactive oxygen species and reactive nitrogen species [9]. The thiol group of glutathione can cleave the covalent modification of Mpro induced by Mpro inhibitors. These observations suggest that these inhibitors may be susceptible to inactivation within living cells. Consequently, it is crucial to evaluate the efficacy of Mpro inhibitors in cell-based assays to ensure their effectiveness as therapeutic agents.

Evaluating the efficacy of Mpro inhibitors in suppressing SARS-CoV-2 replication presents significant challenges for most laboratories. Standard cellular assays for this purpose require the use of infectious viral particles, necessitating a Biosafety Level 3 (BSL-3) containment facility. These BSL-3 facilities require specialized equipment typically unavailable in most research settings and pose a potential risk of infection for personnel conducting the experiments. Additionally, expertise in handling such hazardous materials is essential for safe and reliable assay execution.

Among selenium-containing compounds, selenoneine stands out for its unique biochemical properties attributed to the presence of the metalloid selenium within its structure [10]. Derived from histidine betaine, selenoneine incorporates a selenium atom into its imidazole ring, forming a distinct imidazole-2-selone structure. This unique compound has been identified primarily in marine organisms and individuals with a diet rich in fish [11,12,13,14]. Ergothioneine, a sulfur-containing compound, is structurally analogous to selenoneine, with the key difference being a sulfur atom replacing selenium within the structure. Both selenoneine and ergothioneine are biosynthesized by fungi and other microbes and subsequently accumulate in plants and animals [15]. Given the inhibitory effect of ebselen, a molecule containing a selenazole ring, on Mpro activity [3], we hypothesize that selenoneine, with its structurally similar imidazole-2-selone moiety, may also possess inhibitory potential against Mpro. However, the impact of selenoneine on Mpro activity remains unexplored.

To overcome the limitations of BSL-3 assays for evaluating Mpro inhibitors, we established a novel cellular assay suitable for BSL-1 laboratories. This user-friendly method utilizes standard equipment and conventional laboratory techniques, eliminating the biosafety risks associated with infectious viral particles. In this assay, Mpro is expressed in HEK293 or COS-1 cells, incorporating N-terminal glutathione *S*-transferase (GST) and C-terminal hemagglutinin (HA) tags for efficient detection and manipulation. The inhibitory effect of candidate compounds on Mpro activity was assessed using a conventional anti-HA Western blot. Our method is simple and inexpensive, making it readily usable in standard laboratories, even by researchers with limited experience in infectious diseases. Utilizing this assay, we investigated the efficacy of GC376, boceprevir, carmofur, ebselen, and selenoneine.

## 2. Results

### 2.1. Development of a Cellular Assay for Evaluating Mpro Activity

**Figure 1:**
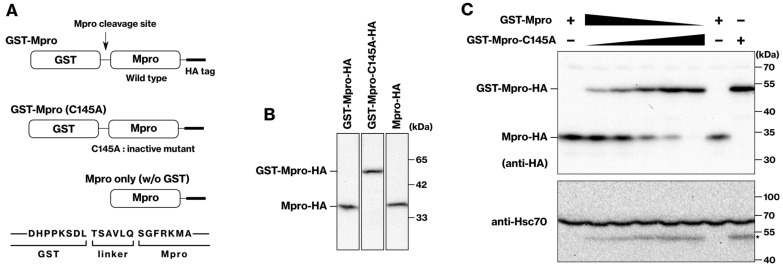
**Autodigestion of GST-Mpro-HA protein.** (**A**) Schematic representation of the GST-Mpro-HA fusion protein. The protein construct includes N-terminal glutathione *S*-transferase (GST) and C-terminal hemagglutinin (HA) tags flanking the Mpro domain. A cleavage site was engineered at the junction between GST and Mpro to enable autodigestion. The C145A mutation within Mpro disrupts its protease activity. (**B**) HEK293 cells were transfected with plasmids encoding GST-Mpro-HA, GST-Mpro-C145A-HA, or Mpro-HA. Cell lysates were prepared 24–30 h post-transfection and analyzed by SDS-PAGE/immunoblotting with anti-HA antibody. All lanes are from the same blot. (**C**) The GST-Mpro vector was diluted with the GST-Mpro-C145A vector, and the cells were transfected with varying ratios (1:10, 1:20, 1:50, 1:100, 1:500) of GST-Mpro-HA and its protease-deficient GST-Mpro-C145A-HA mutant. The asterisk marks the GST-Mpro-HA protein band.

To assess Mpro activity in our cellular assay, we monitored the autodigestion of the GST-Mpro construct expressed in mammalian cells. In a previous work describing the purification of untagged Mpro protein from SARS-CoV-1 [16], SARS-CoV-1 Mpro was N-terminally tagged with GST, and GST was subsequently removed by the endoprotease activity of Mpro in a self-digestive manner. Here, we present an improved approach for SARS-CoV-2 Mpro. We utilize a GST tag fused to Mpro via a linker sequence specifically cleavable by Mpro itself (Figure 1A). This cleavable linker design offers a significant advantage by enabling the self-removal of the GST tag after protein expression within the cells. HEK293 cells were transfected with a plasmid expressing GST-Mpro-HA. A Western blot analysis using anti-HA antibodies revealed a protein band at approximately 35 kDa (Figure 1B). This band corresponds to Mpro-HA, indicating successful cleavage of the GST tag by Mpro. In contrast, GST-Mpro-C145A retained the GST tag and migrated at approximately 55 kDa. Mpro-HA, not conjugated with GST, migrated at 35 kDa. These results indicate that GST-Mpro-HA underwent successful expression and autocatalytic cleavage of GST from the tagged Mpro-HA construct.

To assess inhibitor effects on Mpro activity, we co-transfected HEK293 cells with a mixture of plasmids expressing wild-type and C145A mutant GST-Mpro. This approach modulates the ratio of digested (35 kDa, Mpro-HA) and undigested (55 kDa, GST-Mpro-HA) protein bands, allowing us to monitor the inhibitor-mediated reduction in Mpro activity. While the C145A mutation abrogates the catalytic activity, it remains susceptible to cleavage by co-expressed wild-type Mpro (Figure 1C). To modulate the ratio of active and inactive Mpro proteins, we co-transfected HEK293 cells with mixtures of wild-type and C145A mutant plasmids at varying ratios (1:10 to 1:500). As expected, a Western blot analysis using an anti-HA antibody revealed the presence of both digested and undigested bands in the anti-HA blot (Figure 1C). The intensity ratio of these bands reflected the specific ratio of the co-transfected plasmids. In our experiment, a 1:10 ratio of wild-type to C145A mutant plasmids was chosen for optimal sensitivity.

### 2.2. Cellular Activity of Mpro Inhibitors: GC376 and Boceprevir

**Figure 2:**
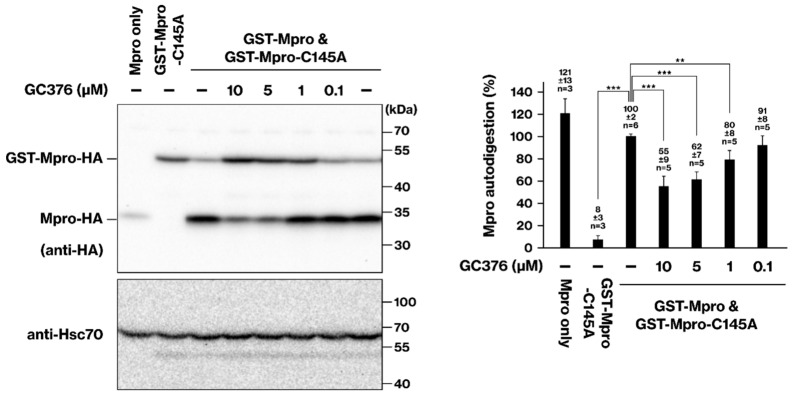
**Inhibition of Mpro activity by GC376 in HEK293 cells.** To assess the inhibitory effect of GC376 on Mpro activity, HEK293 cells were co-transfected with plasmids expressing GST-Mpro-HA (wild type) and GST-Mpro-C145A-HA (protease-deficient mutant) in a 1:10 ratio. After 6–8 h, the culture medium was replaced, and the cells were incubated with GC376 for 16–18 h. Cell lysates were prepared, and Mpro protein levels were analyzed by SDS-PAGE and immunoblotting. The data represent the results of three independent experiments. ***, *p* < 0.001. **, *p* < 0.01.

Our cellular assay was employed to evaluate the effect of GC376 on Mpro activity. For this purpose, the mixture ratio of wild-type and C145 plasmids was adjusted to a 1:10 ratio to optimize the sensitivity of our assay for evaluating GC376. GC376 exhibited a dose-dependent inhibition of Mpro, with increasing inhibitory effects observed at concentrations of 1, 5, and 10 μM (Figure 2). This finding is consistent with a previous report indicating that GC376 inhibited Mpro with the same efficiency in the presence and absence of DTT, suggesting that GC376 was unaffected by the reducing environment [7]. A previous study has reported a GC376 IC50 of 0.03 μM in a cell-free assay [6]. In contrast, our cellular assay identified a 100-fold higher concentration for optimal Mpro inhibition as a semi-quantitative result (Figure 2). This is consistent with the reported EC50 of GC376, which is 3.37 μM, the concentration required to inhibit cytotoxicity caused by SARS-CoV-2 infection.

**Figure 3:**
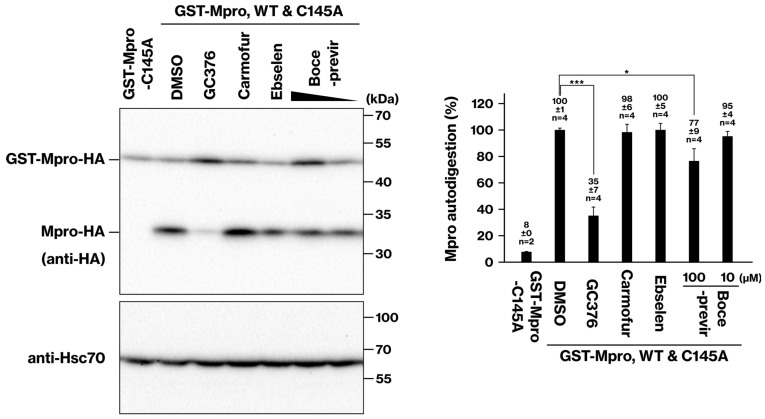
**Effects of boceprevir, carmofur, and ebselen on Mpro activity in HEK293 cells.** Similarly to Figure 2, cells were co-transfected with Mpro plasmids and treated with carmofur (100 μM), boceprevir (100 μM and 10 μM), ebselen (100 μM), or GC376 (10 μM, positive control). Cell lysates were analyzed by SDS-PAGE and immunoblotting. Data from two independent experiments are shown. ***, *p* < 0.001. *, *p* < 0.05.

We further evaluated the efficacy of boceprevir using our cellular assay. Boceprevir inhibited Mpro activity in a dose-dependent manner but at a concentration (100 μM) 10 to 100 times higher than that of GC376 (Figure 3). Interestingly, this observed difference aligns well with the reported IC50 values of boceprevir (4.13 μM) and GC376 (0.03 μM) in a cell-free assay [6]. This consistency suggests that our cellular assay effectively reflects the intrinsic inhibitory potential of Mpro inhibitors observed in cell-free systems.

### 2.3. Deactivation of Mpro Inhibitors Ebselen and Carmofur in a Cell-Based Assay

We evaluated the potential inhibitory effects of carmofur and ebselen on Mpro activity using our cellular assay. In contrast to GC376 and boceprevir, neither carmofur nor ebselen exhibited any significant inhibitory effect on Mpro at the tested concentrations (Figure 3). Thus, while carmofur and ebselen showed IC50 values suggesting potential for Mpro inhibition in cell-free assays (0.2 μM and 3.7 μM, respectively) [6,7], they did not exhibit inhibitory activity in our cellular assay. It is important to note that a previous study reported the inactivation of carmofur and ebselen by DTT, a reducing agent commonly used in protein purification [7]. This observation suggests that the reducing cellular environment might be responsible for deactivating carmofur and ebselen, thereby abrogating their potential inhibitory effects on Mpro.

### 2.4. Noncovalent Binding Energy Calculations for GC376, Boceprevir, and Carmofur

**Figure 4:**
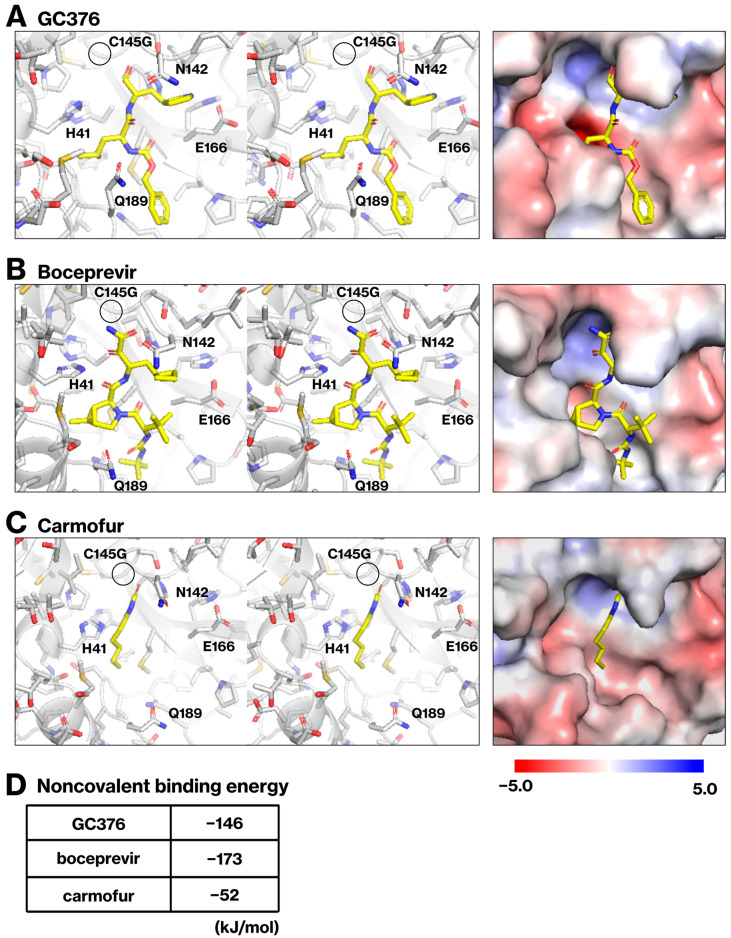
**Computational analysis of noncovalent binding energies between Mpro and inhibitors.** Panels (**A**–**C**) illustrate the optimized structures of the Mpro C145G mutant bound to GC376 (**A**), boceprevir (**B**), and carmofur (**C**). The C145G mutation is indicated by circles. The right panels show the van der Waals surfaces and surface charge distributions of the complexes (red, −5.0 kT/e to blue, +5.0 kT/e). The table in (**D**) displays the calculated binding energies for the noncovalent interactions, obtained using the fragment molecular orbital method implemented in GAMESS with DFTB3, PCM, and DFT-D3(BJ).

GC376 and boceprevir bind to the active site of Mpro through a complex network of hydrogen bonds, ensuring a structural complementarity [6,17]. We hypothesized that the noncovalent interactions between Mpro and various inhibitors may be important for their in vivo activity and performed binding energy calculations. While the reported crystal structures of Mpro/inhibitor complexes revealed covalent bonds formed between C145 of Mpro and GC376, boceprevir, and carmofur [3,6,17], we computationally analyzed noncovalent interactions by replacing C145 with glycine (Figure 4A–C). We employed the fragment molecular orbital method to calculate the binding energies of noncovalent interactions between Mpro and the inhibitors (Figure 4D). GC376, boceprevir, and carmofur exhibited binding energies of −146 kJ/mol, −173 kJ/mol, and −52 kJ/mol, respectively. Interestingly, carmofur, which lacked an inhibitory effect in the cellular assay (Figure 3), had the lowest binding energy. This observed correlation between binding energy and inhibitory activity suggests that the strength of noncovalent interactions between Mpro and inhibitors may be a key factor influencing their efficacy.

### 2.5. Selenoneine as a Potential Mpro Inhibitor

**Figure 5:**
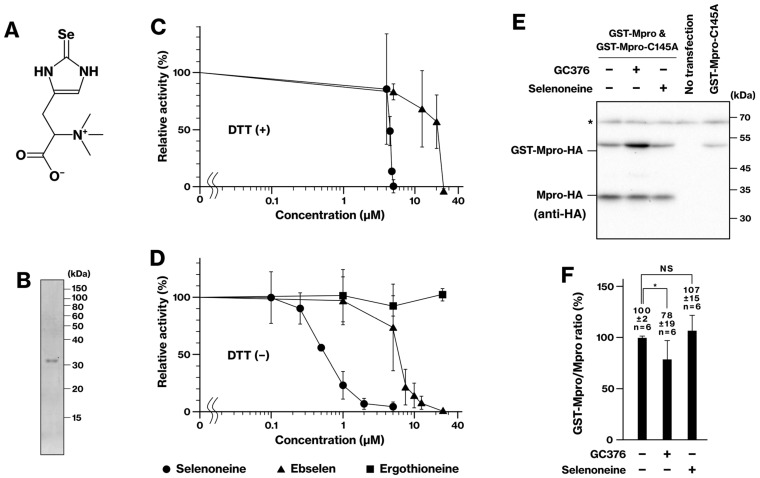
**Selenoneine inhibits Mpro in the cell-free but not in the cell-based assay.** (**A**) Chemical structure of selenoneine. (**B**) Purified GST-Mpro protein visualized by SDS-PAGE. SARS-CoV-2 Mpro was expressed with N-terminal GST and C-terminal His tags (GST-Mpro-6His) in bacteria. The protein was purified by immobilized metal ion affinity chromatography followed by tag cleavage using autodigestion and HRV 3C protease. (**C**,**D**) Inhibition of Mpro activity by selenoneine, ebselen, and ergothioneine (100 μM) in the cell-free assay, with (**C**) and without (**D**) the reducing agent DTT, measured by a fluorescent substrate. (**E**,**F**) HEK293 or COS-1 cells were transfected and treated with selenoneine (100 μM) or GC376 (positive control, 10 μM). The graph depicts data from three independent experiments. In panel E, the asterisk indicates a non-specific band, and all lanes were obtained from COS-1 cells. *, *p* < 0.05. NS, not significant.

We next evaluated the inhibitory effect of selenoneine (Figure 5A) on the purified Mpro activity (Figure 5B). Initially, we assessed its activity in the presence of DTT, a reducing agent carried over from the Mpro purification process. Interestingly, selenoneine exhibited inhibitory activity against Mpro in the cell-free assay, with a potency surpassing that of ebselen (Figure 5C). We further investigated the inhibitory activity of selenoneine in the absence of the reducing agent. For this purpose, we dialyzed the purified protein to remove DTT prior to the cell-free assay. Interestingly, selenoneine exhibited significantly enhanced inhibitory activity against Mpro in the absence of DTT, requiring a 10-fold lower concentration compared to the DTT-containing assay (Figure 5C,D). This observation suggests that selenoneine, like ebselen (whose inhibitory effect was also slightly enhanced, Figure 5D), is susceptible to reducing agents such as DTT. Ergothioneine did not inhibit Mpro in the presence and absence of DTT (Figure 5D). We further evaluated the potential inhibitory effects of selenoneine in our cellular assay. Similarly to the negative results observed for ebselen and carmofur, selenoneine did not display any inhibitory effect on Mpro at the tested concentrations (10 and 100 μM) (Figure 5E,F). This reinforces the notion that inhibitors susceptible to DTT in cell-free assays, including ebselen and carmofur [7], might not translate into effective Mpro inhibitors within the cellular context owing to potential inactivation in the reducing environment.

## 3. Discussion

A previous study has demonstrated that some Mpro inhibitors lose efficacy in the presence of reducing agents, suggesting potential non-specific inhibition through covalent modification at the catalytic C145 residue [7]. Namely, these inhibitors target the C145 residue, leading to the covalent modification that can be cleaved in a reducing environment. Within a cellular context, reducing agents such as glutathione can cleave these modifications. While the inhibition of Mpro by these inhibitors has been observed in cell-free assays, their efficacy in cellular environments may vary. This raises concerns about the generalizability of data obtained in cell-free assays, highlighting the need for inhibitor evaluation within the cellular context. However, conventional cellular assays often require BSL-3 facilities, limiting access for many laboratories. To address this challenge, we develop a convenient and safe BSL-1-compatible cellular assay to investigate Mpro inhibitor activity.

Our cellular assay demonstrates the ability to assess the specific inhibitory activity of Mpro inhibitors. GC376 exhibited a dose-dependent inhibition of Mpro activity (Figure 2), underscoring the semi-quantitative nature of the assay. Furthermore, the comparison of GC376 and boceprevir closely aligns with the IC50 values reported in cell-free assays (Figure 3). The lack of inhibitory activity observed for carmofur and ebselen (Figure 3) is consistent with a previous report demonstrating their inactivation by the reducing agent DTT [7]. A previous study demonstrated the nuclear localization of Mpro [18]. Since GST lacks nuclear localization signals and nuclear export signals, we speculate that the localization of the GST-Mpro protein may be similar to that of Mpro.

Our findings, along with previous reports, suggest that the in vivo efficacy of covalent Mpro inhibitors may not solely depend on covalent bond formation. Compounds with sufficiently stable noncovalent interactions alongside covalent modification might retain activity within the cellular environment. For instance, a previous study demonstrated that covalent inhibitors, such as carmofur and ebselen, non-specifically inhibit Mpro in a cell-free assay and are therefore deactivated under reducing conditions [7]. On the other hand, in the co-crystal structure of GC376 and Mpro, GC376 covalently binds to the catalytic C145 residue of Mpro [6]. Boceprevir, originally developed for hepatitis C virus nonstructural 3 (NS3) protease inhibition, covalently modifies both the targeted NS3 serine residue [5] and the C145 residue of Mpro as observed in the co-crystal structure [17]. Similarly, nirmatrelvir, but not ensitrelvir, targets the C145 residue of Mpro [1,8]. These results indicate that not all covalent inhibitors were inactivated in vivo. Our FMO calculations revealed higher binding energies for GC376 and boceprevir than for carmofur (Figure 4), suggesting stronger noncovalent interactions with Mpro. This finding aligns with previous molecular dynamic simulations demonstrating the superior stability of GC376 within the Mpro active site compared to carmofur and ebselen [7]. These combined results suggest that the robust noncovalent interactions of GC376 and boceprevir contribute to their stability, potentially hindering the reductive cleavage of the covalent bond with C145 and ensuring their in vivo efficacy.

Interestingly, a previous study reported that ebselen inhibited viral replication in a cellular assay [3]. However, our findings demonstrate that ebselen does not inhibit Mpro activity in a reducing environment, as observed with DTT in a cell-free assay [7] and likely within our cellular assay (Figure 5E,F). This discrepancy suggests that ebselen might possess an alternative mechanism for inhibiting viral replication that is independent of Mpro activity. Indeed, ebselen and its derivatives have been reported to inhibit the N7-methyltransferase activity involved in viral RNA cap modification [19,20]. Future studies are warranted to elucidate these potential alternative mechanisms.

Our cell-free assay revealed a remarkable difference between selenoneine and its sulfur analog ergothioneine. While selenoneine inhibited Mpro activity (Figure 5C,D), ergothioneine lacked any inhibitory effect (Figure 5D). This suggests that the inhibitory activity relies not only on electrostatic interaction and hydrogen bonds but also on the covalent interaction between the C145 residue and selenium. Since selenoneine displayed stronger inhibitory activity than ebselen (Figure 5C,D), this compound is a promising candidate for the development of Mpro inhibitors.

The major benefit of our current assay is its ease of implementation, requiring no specialized equipment. Another advantage of our system is its capability to tailor the assay sensitivity to various inhibitors by manipulating the mixture ratio of wild-type and C145 mutant plasmids (Figure 1C). In the case of Figure 2, the mixture ratio was specifically adjusted to a 1:10 ratio to suit GC376. However, its major drawback lies in its throughput. While previous studies have utilized fluorescence-based high-throughput assays [1,21], ours relies on Western blotting and has a low throughput. Conversely, the high-throughput methods necessitate plate readers capable of fluorescence detection or automated fluorescence microscopes with analysis software. However, such systems are expensive and inaccessible to many researchers. Since our method employs Western blot, it can be conducted in standard laboratory settings, making it suitable for initial experiments testing the Mpro inhibitors within cells.

Our cellular assay employs a GST-Mpro-HA plasmid transfected into mammalian cells, followed by the evaluation of inhibitor activity using a conventional anti-HA Western blot. This approach bypasses the use of live viral particles and allows for safe BSL-1 containment. This key advantage eliminates the need for specialized facilities and expertise in the assay for infectious diseases, making the assay readily accessible to researchers in various fields, including biochemists and organic chemists. The accessibility of our strategy allows a wider range of scientists to directly evaluate the Mpro inhibitory potential of their compounds within a cellular context.

## 4. Materials and Methods

### 4.1. Plasmids

For Mpro expression, the coding sequence (YP_009725301.1) corresponding to bases 10055-10972 of the SARS-CoV-2 (NC_045512) genome was synthesized (Twist Bioscience, South San Francisco, CA, USA) and cloned downstream of a GST tag with a preceding Mpro cleavage sequence (TSAVLQ↓SGFRK) (arrow indicates the cleavage site). A C-terminal fusion containing six histidines (6His) and three HA (3HA) tags was appended to Mpro. This cassette was then cloned into a pTwist CMV BetaGlobin vector, resulting in the GST-Mpro-6His-3HA vector. Additionally, a Mpro C145A mutant and a construct encoding Mpro-6His-3HA without the N-terminal GST tag were generated.

### 4.2. Transfection and Western Blot

Both HEK293 and COS-1 cells were cultured in Dulbecco’s modified Eagle medium high glucose (DMEM, Sigma-Aldrich, St. Louis, MO, USA, Cat. No. D5796) at a density of 3.6 × 10^5^ cells per well in a 24-well plate (Corning Inc., Corning, NY, United States, Cat. No. 3526) pretreated with bovine serum overnight. HEK293 cells were grown in DMEM high glucose (Sigma-Aldrich, St. Louis, MO, USA, Cat. No. D5796) supplemented with 5% FBS, while COS-1 cells received DMEM containing a mixture of 5% bovine serum and 1% FBS. For transfection, the cells were plated and incubated with a mixture of 194 ng of plasmid DNA (1:10–1:500 GST-Mpro WT:C145A) and 0.97 μL of Lipofectamine 2000 (Life Technologies, Carlsbad, CA, USA, Cat. No. 11668027) following the manufacturer’s instructions. After 24–36 h, the cells were lysed with Laemmli SDS-PAGE sample buffer.

For Mpro inhibitor treatment, the medium was replaced with fresh media containing the inhibitor 6–8 h post-transfection. Cells were incubated with the inhibitors (GC376, Selleck Chemicals, Cat. No. S0475; Boceprevir, Selleck Chemicals, Houston, TX, USA, Cat. No. S3733; Carmofur, LKT Laboratories, Cat. No. C0174; Ebselen, Tokyo Chemical Industry, Tokyo, Japan, Cat. No. E0946) for 16–18 h before preparation of SDS lysates.

Proteins were separated by SDS-PAGE and transferred to a 0.2 μm PVDF membrane (FluoroTrans, FUJIFILM Wako, Osaka, Japan, Cat. No. 365-00681 or Cytiva, Tokyo, Japan, Cat. No. BSP0161). Following blocking with 5% skim milk/TBS-T/2 mM EDTA, the membrane was probed with anti-HA (Life Technologies, Cat. No. 715500) and anti-Hsc70 (Santa Cruz Biotechnology, Dallas, TX, USA, Cat. No. sc-7298) antibodies. Primary antibodies were diluted with ImmunoEnhancer (FUJIFILM Wako, Osaka, Japan, Cat. No. 290-68603), while secondary antibodies were diluted in 5% skim milk/TBS-T. Following incubation, the signal intensities of GST-Mpro and Mpro were detected and quantified using the ChemiDoc XRS Plus system (Bio-Rad Laboratories, Hercules, CA, United States). Cleavage efficiency was calculated as the ratio of GST-Mpro to total protein (GST-Mpro + Mpro) and normalized to the DMSO control. Data were presented as the mean ± standard deviation. Statistical significance was determined using Student’s or Welch’s *t*-test (samples prepared singly or in duplicate). In Figure 5E,F, one of the three independent repeats involved HEK293 cells, and the remaining two utilized COS-1 cells.

### 4.3. Binding Energy Calculation

To calculate the binding energy of Mpro inhibitors, we employed the co-crystal structures of Mpro with GC376 (PDB 6WTT) [6] and carmofur (PDB 7BUY) [3]. In these structures, GC376 and carmofur formed a hemithioacetal at the C145 residue. For noncovalent interaction calculations, we mutated Mpro C145 to glycine, converted the hemithioacetal into an aldehyde, and added hydrogen atoms using the H++ server [22].

The fragment molecular orbital (FMO) method [23] was employed to calculate the noncovalent binding energy between Mpro and the inhibitors. Calculations were performed using GAMESS 2022R.2 [24,25] with input files generated by Facio [23]. We utilized the third generation of the density functional tight binding (DFTB3) method [26] with the 3OB-3-1 parameter set [27,28] for orbital calculations. The conductor-like polarizable continuum model (PCM) [26] was employed to calculate the potential energy in the water phase, incorporating dispersion correction using the modified third implementation of Grimme’s empirical dispersion correction, DFT-D3(BJ) [29]. Prior to free energy calculations, both Mpro and inhibitor structures were optimized over 2000 steps using a Hessian update method. The dispersion-corrected free energy in solvent for the Mpro/inhibitor complex (*E_complex_*) and Mpro alone (*E_Mpro_*) was then calculated with the three-body expansion of the FMO method [24]. The free energy of inhibitors (*E_inhibitor_*) was calculated using the same DFTB3, PCM, and DFT-D3(BJ) method employed for the Mpro/inhibitor complex. Binding energy (*ΔE_bind_*) was then determined as follows: *E_complex_* − (*E_Mpro_* + *E_inhibitor_*). To visualize the electrostatic potential, PDB2PQR 2.1.1 [30] and Adaptive Poisson–Boltzmann Solver 3.0.0 [31] were used to calculate the potential on Mpro’s solvent-accessible surface. The molecular surface was generated using PyMOL 2.5.0 [32].

### 4.4. Purification of Recombinant Mpro Protein

We constructed the Mpro expression cassette as previously described [16]. Briefly, the SARS-CoV-2 Mpro coding sequence was cloned into a pET29b vector containing N-terminal GST and C-terminal 6His tags. The GST tag was separated from Mpro by the Mpro cleavage sequence, and the human rhinovirus (HRV) 3C protease cleavage sequence was located before the C-terminal 6His tag.

Recombinant GST-Mpro-6His protein expression was achieved in BL21(DE3) *Escherichia coli* transformed with pET29b/GST-Mpro-6His. Details of protein expression and purification steps are provided elsewhere [33,34]. Briefly, the bacteria were cultured in LB medium at 37 °C to mid-log phase. Expression of the recombinant protein was then induced with 0.5 mM IPTG for 3−4 h at 37 °C, followed by bacterial collection through centrifugation. Following resuspension in 10 packed cell volumes of buffer (20 mM NaPi, pH 6.8, 300 mM NaCl), the cells were lysed using a combined approach: enzymatic digestion with 1 mg/mL lysozyme on ice for 15 min, followed by three freeze–thaw cycles and sonication with a sonicator (UR-20P, Tomy Digital Biology, Tokyo, Japan). The lysate was then supplemented with 0.1% Triton X-100 and clarified by centrifugation. Finally, bacterial cleavage of the N-terminal GST tag was verified.

The recombinant Mpro-6His protein was purified using Talon resin (TaKaRa Bio, Shiga, Japan, Cat. No. 635501). Briefly, the lysate was incubated with the resin for 30 min at 4 °C with rotation, followed by washes with buffer containing 20 mM NaPi (pH 6.8), 300 mM NaCl, 0.1% Triton X-100, and 10 mM imidazole. Mpro-6His was eluted from the Talon resin using a wash buffer supplemented with 300 mM imidazole. The eluted protein was then dialyzed overnight at 4 °C against a buffer containing 50 mM Tris-HCl (pH 7.5), 150 mM NaCl, 0.01% Triton-X-100, 10% glycerol, 0.5 mM EDTA, and 0.5 mM DTT using a 10 kDa molecular weight cut-off Slide-A-Lyzer dialysis cassette (ThermoFisher, Waltham, MA, USA, Cat. No. 66380).

To remove the His tag, 1.4 mg of Mpro-6His protein was digested with 38 U of HRV 3C protease (TaKaRa Bio, Shiga, Japan, Cat. No. 7360) at 4 °C for 16 h. The cleaved protein was then isolated by incubating the digest with Ni-NTA agarose (FUJIFILM Wako, Osaka, Japan, Cat. No. 143-09763) at 4 °C for 1 h. The unbound fraction was collected and dialyzed overnight at 4 °C against a storage buffer containing 20 mM Tris-HCl (pH 7.5), 100 mM NaCl, 0.01% Triton X-100, 50% glycerol, 1 mM EDTA, and 1 mM DTT. The protein concentration was quantified through the Bradford assay using Bio-Rad protein dye reagent (Bio-Rad Laboratories, Hercules, CA, USA Cat. No. 5000006).

### 4.5. Preparation of Selenoneine

Selenoneine was purified from the lysate of genetically engineered *Aspergillus sojae* (Kikkoman Corporation, Chiba, Japan) using preparative HPLC. The filtered aqueous extract (5 mL aliquots) was injected onto a TSKgel ODS-100V column (TOSOH, Tokyo, Japan; 28 mm i.d. × 150 mm, 5 μm), and eluted with 0.1% formic acid. The eluent was monitored at 220 nm for selenoneine detection. Purity analysis was performed using analytical HPLC with a Symphonia C18 column (JASCO, Tokyo, Japan; 4.6 mm i.d. × 150 mm, 5 μm) and 0.1% formic acid as described previously [33].

### 4.6. Cell-Free Mpro Protease Assay

The effect of selenoneine on Mpro’s protease activity was evaluated using a fluorogenic peptide substrate, Ac-Abu-Tle-Leu-Gln-MCA (Peptide Institute, Osaka, Japan, Cat. No. 3250-v). The reaction mixture contained 50 mM Tris-HCl (pH 7.4), 10 μM substrate, 2 μg/mL Mpro, and varying concentrations of ebselen, selenoneine, or ergothioneine (Nacalai Tesque, Kyoto, Japan). Tris-HCl and the substrate were preincubated at 37 °C for 5 min in a reaction chamber. Mpro and varying concentrations of test compounds (ebselen, selenoneine, or ergothioneine) were then added, followed by incubation for 20 min at 20 °C. Fluorescence intensity was then measured using a fluorescence spectrophotometer (F-7000, HITACHI, Tokyo, Japan) with excitation and emission wavelengths set at 380 nm (10 nm slit) and 460 nm (10 nm slit), respectively. This resulted in a final DTT concentration of approximately 500 nM owing to carry-over from the storage buffer. To remove DTT for reactions, the purified Mpro protein was dialyzed against a buffer identical to the Mpro storage buffer but lacking DTT. Dialysis was performed using an Xpress Micro Dialyzer (3.5 kDa molecular weight cut-off; Scienova, Thuringia, Germany, Cat. No. 40782) for 2 h with buffer changes every 30 min.

## 5. Patents

The findings presented in this study, particularly the investigation of selenoneine, ebselen, and ergothioneine’s effects on Mpro in a cell-free assay, are related to the following patent applications: N. Suzuki et al. Publication No. WO/2022/177029 [35] (25 August 2022), International Application No. PCT/JP2022/007397 (22 February 2022), World Intellectual Property Organization, and N. Suzuki et al. Application No. 2021-165803 (7 October 2021), Japan Patent Office. 

## Data Availability

Dataset available on request from the authors.

## Abbreviations

BSL	biosafety level
DMEM	Dulbecco’s modified Eagle medium
GST	glutathione *S*-transferase
HA	hemagglutinin
Mpro	main protease
ORF	open reading frame
FMO	fragment molecular orbital
DFTB3	third generation of the density functional tight binding
PCM	polarizable continuum model
